# Association of newly identified genetic variant rs2853677 of *TERT* with non-small cell lung cancer and leukemia in population of Jammu and Kashmir, India

**DOI:** 10.1186/s12885-019-5685-2

**Published:** 2019-05-24

**Authors:** Gh. Rasool Bhat, Amrita Bhat, Sonali Verma, Itty Sethi, Ruchi Shah, Varun Sharma, Khursheed A. Dar, Deepak Abrol, Subiya Kaneez, Sandeep Kaul, Ramesh Ganju, Rakesh Kumar

**Affiliations:** 1 Cancer Genetics Research Group, School of Biotechnology, Shri Mata Vaishno Devi University, Katra, India; 2 Human Genetics Research Group, School of Biotechnology, Shri Mata Vaishno Devi University, Katra, India; 3 Chest Disease Hospital, Government Medical College, Srinagar, India; 4 Department of Radiotherapy, Government Medical College, Jammu, India; 5 Department of Radiotherapy, Government Medical College, Srinagar, India; 6 Department of surgical Oncology, Shri Mata Vaishno Devi Narayana Super speciality hospital, kata, India; 7 Department of Pathology, College of Medicine, The OHIO State University, Columbus, USA

**Keywords:** Non-small cell lung cancer, Leukemia, Telomerase reverse transcriptase *(TERT)*, Jammu and Kashmir

## Abstract

**Background:**

Telomere genetics has recently been emerged as an important field in molecular oncology. Various genome-wide association studies in different population groups have revealed that polymorphisms in Telomere maintenance gene (*TERT*) gene located on 5p15.33 is associated with susceptibility to leukemia and lung cancer risk. However, association of *TERT* with leukemia and lung cancer risk in north Indian population groups is still unknown. This study observed the association between genetic variant rs2853677 of *TERT* and leukemia and lung cancer in the state of Jammu and Kashmir, India.

**Methods:**

A total of 781 subjects, out of which 381 cases (203 leukemic patients and 178 non-small cell lung cancer patients NSCLC) and 400 healthy controls were recruited for the study. Genetic variant rs2853677of *TERT* was detected using the real-time and Taqman Chemistry. Hardy-Weinberg Equilibrium was assessed using the chi square test. The allele and genotype- specific risks were estimated as odds ratio with 95% confidence interval.

**Results:**

We observed that variant rs2853677 was strongly associated with lung cancer and leukemia risk with an odds ratio (OR) =1.8 (1.03–3.2 at 95% CI); *p* value (adjusted) = 0.03; odds ratio (OR) =2.9 (1.4–5.5.at 95% CI); p value (adjusted) = 0.002, respectively.

**Conclusion:**

The results of this study suggested that rs2853677 of *TERT* signifies association in multiple cancers and suggests that it can become potential marker for diagnosis of non-small cell lung cancer and leukemia. The study will provide an insight in understanding the genetic etiology and highlights the role of telomere-associated pathways in non-small cell lung cancer and leukemia. However, it would be quite interesting to explore the contribution of this variant in other cancers as well.

**Electronic supplementary material:**

The online version of this article (10.1186/s12885-019-5685-2) contains supplementary material, which is available to authorized users.

## Background

Lung cancer has highest mortality rate among cancers for both males and females, whereas males are more affected than females in leukemia by the ratio 1:5:1:0 [1]. Both the cancers are aggressive in nature, and this is one of the major global health concern worldwide. Lung cancer constitutes 12.9% of all newly diagnosed cancer cases and 19.4% of all cancer associated deaths globally [2, 3] took place. Lung Cancer is the major cause of cancer related deaths in India accounting for 63,000 newly diagnosed patients each year with 52,000 deaths, and thereby contributing to 9.3% of all cancer associated deaths [4, 5]. Furthermore, lung cancer in Kashmir region (J&K) was reported to be the second most common malignancy in hospital based study. [6]. While in Jammu region (J&K), unpublished data from our research group found that lung cancer and breast cancer are most commonly occurring cancers followed by esophageal cancer.

Lung cancer and Leukemia are multifactorial in nature, where both genetic and non-genetic factors like environmental pollution, occupational exposure and smoking mainly contribute to its etiology [7]. Single nucleotide polymorphism (SNP) genotyping and Genome wide association studies (GWAS) in Japanese population have revealed that chromosome 5p15.33 is the strong candidate region associated with lung cancer [8]. Genetic variant rs2853677 of telomere maintenance gene *TERT* is located in an intronic region of chromosome 5p15.33 (chr5:1,253,147-1,295,069) [9]. Telomeres are actually caps located at the terminal end of the linear chromosomes, which acts as an important factor in maintaining the genomic stability. In humans, telomeres comprise of guanine rich hexa nucleotide (TTAGGG) repeats. The stability of the telomeres solely depends on the integrity of the associated telomere maintenance proteins, which always work in coordinated manner, referred to as the shelterin complex [10]. Shelterin complex comprises of different factors like Telomeric Repeat Binding Factor 1 (TRF1), Telomeric Repeat Binding Factor 2 (TRF2), TRF1-Interacting Nuclear Factor 2 (TIN2), Protection of Telomeres 1 (POT1), Tripeptidyl Peptidase 1 (TPP1) and Repressor Activator Protein 1 (RAP1) [11]. Among the binding factors TRF1 and TRF2 bind to the double stranded telomeric DNA segment as homodimers. TRF1 inhibits the action of telomerase thus acts as negative regulator of telomere length [12] and the stability of T loop is maintained by TRF2. Mutation in TRF2 results in the activation of Ataxia Telangiectasia Mutated (ATM) protein mediated DNA damage signal which leads to end-to-end telomere fusion and telomere homologous recombination, leading to typical process of telomere sister-chromatid exchange (T-SCE) [13]. However, repressor activator protein 1 (RAP1) directly binds to TRF2 protein which is crucial for efficient binding of TRF2 protein to the telomeric DNA. Another important factor POT1 protects the chromosome ends from fusion and a typical recombination and helps in the recruitment of telomerase to the telomeric DNA, thus contributing to the protection of telomeres [14]. TRF-interacting nuclear factor 2 (TIN2) protein mainly binds with other shelterin proteins like TRF1, TRF2 and TPP1, acting as a critical component of the shelterin complex and bridging all these proteins of the shelterin complex together for efficient working. [12]. Shelterin proteins possibly regulate telomere elongation by enrolling Telomerase, which is a ribonucleoprotein complex consisting of reverse transcriptase (*TERT*) and RNA subunit (*TERC*). Telomerase is inactive and suppressed in most of the normal somatic cells. During the S phase of cell cycle, it gets activated and stimulates the replication process of telomeric DNA, which is not replicated, by DNA polymerase during replication [15].

Molecular alterations in the TERT promoter lead to enhanced expression of telomerase, which maintains telomere length thereby letting the cancer cells to continuously proliferate and inhibiting the programmed cell death and apoptosis [16].

In the present study, we conducted an association study of variant rs2853677 of TERT gene and susceptibility to non-small cell lung carcinoma and leukemia in population of Jammu and Kashmir. The critical reason to identify these associations is to understand the genetic heterogeneity of non-small cell lung cancer and leukemia and possibility of using the newly identified genetic variant of telomere maintenance gene *(TERT)* as prognostic biomarker for diagnosis of non-small cell lung cancer and leukemia.

## Methods

### Ethical statement

The study was approved by the Institutional Ethics Review board (IERB) of Shri Mata Vaishno Devi University (SMVDU) vide IERB Serial No: SMVDU/IERB/16/41 and SMVDU/IERB/16/47. The written consent were taken into account from each participant and all the parameters were recorded in pre-designed Performa.

### Sampling

A total of 781 subjects, out of which 381 Cases (203 leukemic patients and 178 non-small cell lung cancer patients) and 400 healthy controls were recruited for study. All cancer cases were histopathologically confirmed. The genomic DNA was isolated from the blood samples using Qiagen DNA Isolation Kit (Catalogue No. 51206). Agarose gel electrophoresis was used to analyze the quality of genomic DNA and quantification was performed using UV spectrophotometer (Nanodrop).

### Genotyping

Genotyping of variant rs2853677 was performed using allele discrimination assay on MX3005p Agilent Real time PCR platform. Taqman Probe labelled with VIC and FAM (Thermofisher Scientific) and UNG Master Mix (Applied Biosystems, USA) were used for genotyping.

Dilutions of assay were made from 40X concentration to 20X using TE (Tris EDTA buffer) as per recommendations of manufacturer. The Volume of total PCR reaction was 10 μl, comprising of 2.5 μl of Taqman UNG Master Mix, 0.25 μl of probe, 3 μl DNA (5 ng/μl) and 4.25 μl nuclease free water was added to make up the final volume. The Following thermal conditions were adopted; hold for 10 min at 95 °C, then 40 cycles of 95 °C for 15 s and 60 °C for 1 min. All the samples were run in 96-well plate with three no template Control (NTC). The post PCR detection system is used to measure allele specific fluorescence. 93 random samples were picked and re-genotyped for cross validation of genotyping calls and the concordance rate was 100%.

### Statistical analysis

Statistical analysis of the data were completed with the application of SPSS software (v.20; Chicago, IL. Chi square test (χ^2^) was performed and genotypic frequencies were tested for total Hardy-Weinberg equilibrium. Logistic regression was used to estimate OR at 95% confidence interval (CI) and respective level of significance as *p* value.

### Results

In the present case control association study, we made an attempt to replicate the novel genetic variant rs2853677 of *TERT* in the population of Jammu and Kashmir. The change in the nucleotide is g.1287079G > A. The clinical characteristic distribution of the cases and controls in non-small cell lung cancer and leukemia are given in Table 1. We used common control set for non-small cell lung cancer and leukemia in which 70% were males and 30% were females. In non-small cell lung cancer cases 80% were males and 20% were females while in leukemia cases, 60.7% were males and 39.3% were females, stating the prevalence of both the cancers are high among males in the Jammu and Kashmir region. The mean ages for control set were 54.53 (±11.9) years, for the non-small cell lung cancer cases were 61.07 (±9.65) years and for the leukemia cases were 40.5 (±14.67) years.

**Table 1:** Showing Clinical Characteristics of Cases and Controls in non-small cell lung cancer and Leukemia

Characteristics	Cases NSCLC (*n* = 178)	Cases Leukemia (*n* = 203)	Control (*n* = 400)
1. Age* (in years)	61.07 ±9.65	40.5 (±14.67)	54.53 ±11.9
2. Gender (in % and No. of Cases)	M = 80 (*n* = 143)	M = 60.6 (*n* = 123)	M = 70 (*n* = 280)
	F = 20(*n* = 35)	F = 39.3(*n* = 80)	F = 30 (*n* = 120)
3. BMI**	22.4 ±3.89	21.2 (±6.08)	24.37 ±4.85
4. Histological Subtypes	AC 0.63(*n* = 112) SCC 0.33(*n* = 59) UDC 0.04(n = 7)	ALL 0.17(n = 35) AML 0.19(*n* = 39) CML 0.49(*n* = 99) CLL 0.14(n = 28) MDS 0.01(n = 2)	- - - -
5. Smoking (% & No. of Cases)			
Yes	82 (*n* = 146)	56.3(*n* = 114)	18(*n* = 72)
No	18(n = 32)	43.7(*n* = 89)	82(*n* = 328)
6. Alcohol (% and No. of Cases)			
Yes	37(*n* = 66)	6(n = 12)	12.5 (*n* = 50)
No	63(n = 112)	94(*n* = 191)	87.5(*n* = 350)
Guthka (% and No. of Cases)			
Yes	9(*n* = 16)	–	6(*n* = 24)
No	91(*n* = 162)	–	94(*n* = 376)

*age in years and **BMI in kg/m^2^

In the current study allele G is being present more in cases (0.60 (NSCLC); 0.71 (Leukemia)) than in controls (0.53), hence, suggesting that allele G is causing risk. The allele frequency distribution has been summarized in Table 2 and Table 3 for non-small cell lung cancer and leukemia respectively. We observed that genetic allele G of variant rs2853677 of *TERT* is significantly associated with non-small cell lung cancer (*p* value =0.03) and leukemia (p value = 2.6 × 10^− 9^) in the population of Jammu and Kashmir. To observe the maximum effect of allele G, we evaluated the association by using dominant model. The OR observed was 1.8 (1.03–3.2) at 95% CI in non-small cell lung cancer and 2.9 (1.5–5.7) at 95% CI in leukemia corrected for age, gender and BMI.

**Table 2:** Allelic frequency distribution and risk associated with the *TERT* variation in non-small cell lung cancer in Jammu and Kashmir Population

GENE/SNP	R.A	Cases (*n* = 178)	Controls (*n* = 400)	HWE	Allelic OR	*p* Value	Dominant OR*	*p* value*
*TERT*/rs2853677	G	0.60	0.53	0.261	1.3 [1.02–1.7]	0.03	1.8 [1.03–3.2]	0.03

*Corrected for age, gender and BMI

**Table 3:** Allelic frequency distribution and risk associated with the *TERT* variation in leukemia in Jammu and Kashmir Population

GENE/SNP	R.A	Cases (*n* = 203)	Controls (*n* = 400)	HWE	Allelic OR	*p* Value	Dominant OR*	*p* value*
*TERT*/rs2853677	G	0.71	0.53	0.654	2.2 [1.6–2.8]	2.6 × 10^−9^	2.7 [1.4–5.2]	0.002

*Corrected for age, gender and BMI

Furthermore, we have evaluated the variant rs2853677 of TERT gene by applying other genetic models as per the risk allele and the results observed were showing positive association of variant in all the three models in case of Leukemia Table 4 and only dominant was found to be associated with non-small cell lung cancer Table 5 .

**Table 4:** showing the association of variant rs2853677 of *TERT* with leukemia in Jammu and Kashmir Population using different genetic models

Genetic Model	Genotype	Odds Ratio	95% CI	*p* Value
Dominant	GG/AG vs AA	2.9	1.65–5.56	0.001
Additive	–	1.9	1.41–2.66	0.0001
Recessive	GG/AA vs GG	2.1	1.40–3.221	0.0003

*Corrected for Age, Gender and BMI

**Table 5:** showing the association of variant rs2853677 of *TERT* with non- small cell lung cancer in Jammu and Kashmir Population using different genetic models

Genetic Model	Genotype	Odds Ratio	95% CI	*p* Value
Dominant	GG/AG vs AA	1.8	1.03–3.19	0.03
Additive	–	1.4	1.00–2.09	0.05
Recessive	GG/AA vs GG	1.2	0.82–3.19	.279

*Corrected for Age, Gender and BMI

**Fig. 1:**
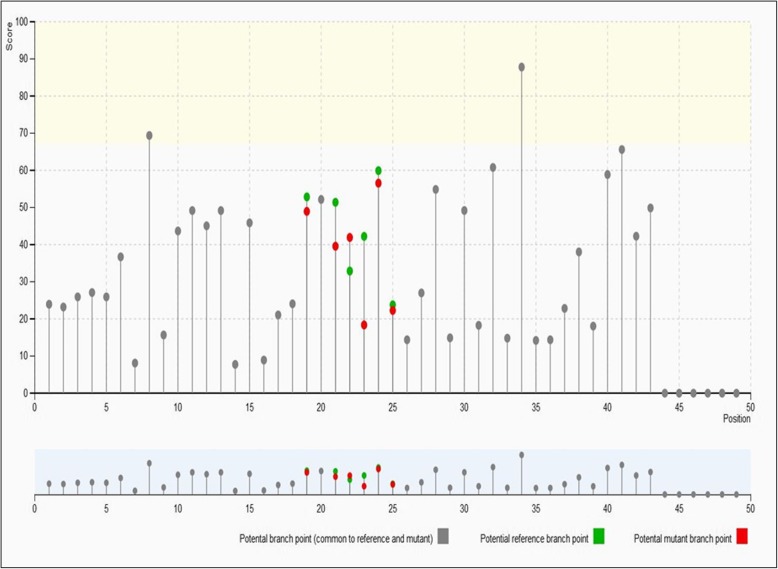
Potential Splice sites in the variation region TERT c.1574-4455C > T. Potential splice site in the variation region of TERTc.1574-4455C > T were described in Fig. 1,whererin green color depicts potential reference branch point and red color indicates potential mutant branch point, grey color for potential branch point (common to reference and mutant)

Smoking, being the primary agent in the etiology of lung cancer in more than 85% of cases. We explored the association of variant rs2853677 of *TERT* gene with smokers only. We selected only those individuals who had history of smoking from more than ten years to check its effect on LC (cases: 147; controls: 70; allelic distribution in Additional file 2: Table S2). The odds ratio observed was 2.4 (1.5–5.2) at 95% CI. *p* value =0.02. Thus, our finding indicates that the risk of variant rs2853677of *TERT* gene was higher in smokers than pooled population (smokers + non-smokers). Furthermore, in order to evaluate the effect of this genetic variant on *TERT* gene using insilco analysis by Human Splicing finder (HSF) [17]. It was observed that exonic splicing enhancer (ESE) site got broken. The majority of the algorithms used for the prediction of enhancer/silencer motifs by HSF (v.3.1) tool as described in Tables 6 and 7 indicated that rs2853677 results in the broken sites for SF2/ASF (IgM-BRCA1) and creation of new binding sites of (serine/arginine rich protein) SRp55 (76.04), SC35 (78.30). Potential splice site in the variation region of TERTc.1574–4455 C > T were described in (Fig. 1), green color depicts potential reference branch point and red color indicates potential mutant branch point, grey color for potential branch point (common to reference and mutant).

**Table 6:**
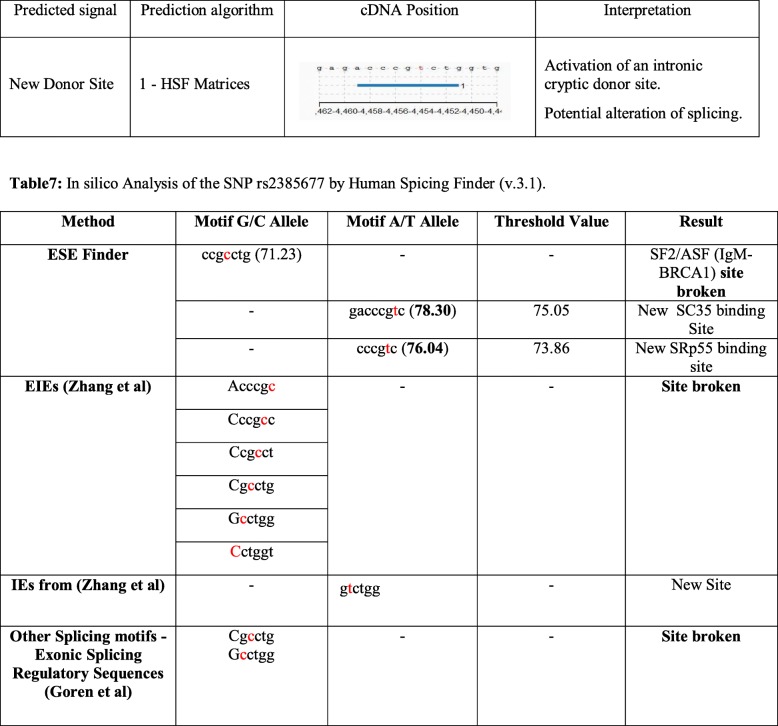
Representation of Potential Alteration of Splicing site by HSF (v.3.1)

**Table 7:** In silico Analysis of the SNP rs2385677 by Human Spicing Finder (v.3.1)

Method	Motif G/C Allele	Motif A/T Allele	Threshold Value	Result
ESE Finder	ccgcctg (71.23)	–	–	SF2/ASF (IgM-BRCA1) **site broken**
	–	gacccgtc (**78.30**)	75.05	New SC35 binding Site
	–	cccgtc (**76.04**)	73.86	New SRp55 binding site
EIEs (Zhang et al)	Acccgc	–	–	**Site broken**
	Cccgcc			
	Ccgcct			
	Cgcctg			
	Gcctgg			
	Cctggt			
IEs from (Zhang et al)	–	gtctgg	–	New Site
Other Splicing motifs - Exonic Splicing Regulatory Sequences (Goren et al)	Cgcctg Gcctgg	–	–	**Site broken**

The values in bold represents the mutant motif threshold value which are higher than the reference motif threshold value indicating their possible role in the alteration of splicing factor binding sites and splicing sites

## Discussion

Genomic wide association studies (GWAS) and candidate gene approach (CGA) have proven to be important tools in understanding genetic complexity and heterogeneity of these multifactorial diseases through case-control association in various ethnic groups. Therefore, this study investigated the association between TERT gene polymorphism and lung cancer and leukemia risk in the less explored population of Jammu and Kashmir of North India. We observed that variant rs2853677 was strongly associated with non-small cell lung cancer and leukemia risk with odds ratio (OR) =1.8 (1.03–3.2 at 95% CI); p value (adjusted) =0.03 in NSCLC and 2.7 (1.4–5.2 at 95% CI); *p* value (adjusted) = 0.002 in leukemia, signifying that TERT polymorphism plays a crucial role in the pathological process of multiple cancers. Our findings are consistent with previous genome wide association studies stating the role of TERT gene in different cancers at different ethnicities [8, 18–22]. Furthermore it was observed that the subtype adenocarcinoma (AC) and squamous cell carcinoma (SCC) of non-small cell lung cancer was found to be associated with odds ratio OR = 1.37 and 1.50 respectively whereas, the subtype undifferentiated carcinoma (UDC) was not significantly associated. The possible reason being small sample size (*n* = 7) However in leukemia, the subtypes Acute Lymphoblastic Leukemia (ALL), Acute Myeloid Leukemia (AML),Chronic Myeloid Leukemia (CML), Chronic Lymphoid Leukemia (CLL) were found to be associated with an OR = 1.75, 1.71, 1.40, 1.81 respectively. Myelodysplastic Syndrome (MDS) subtype of leukemia did not show any association with possible reason of small sample size. Additional file 1: Table S1.

**Fig. 2:**
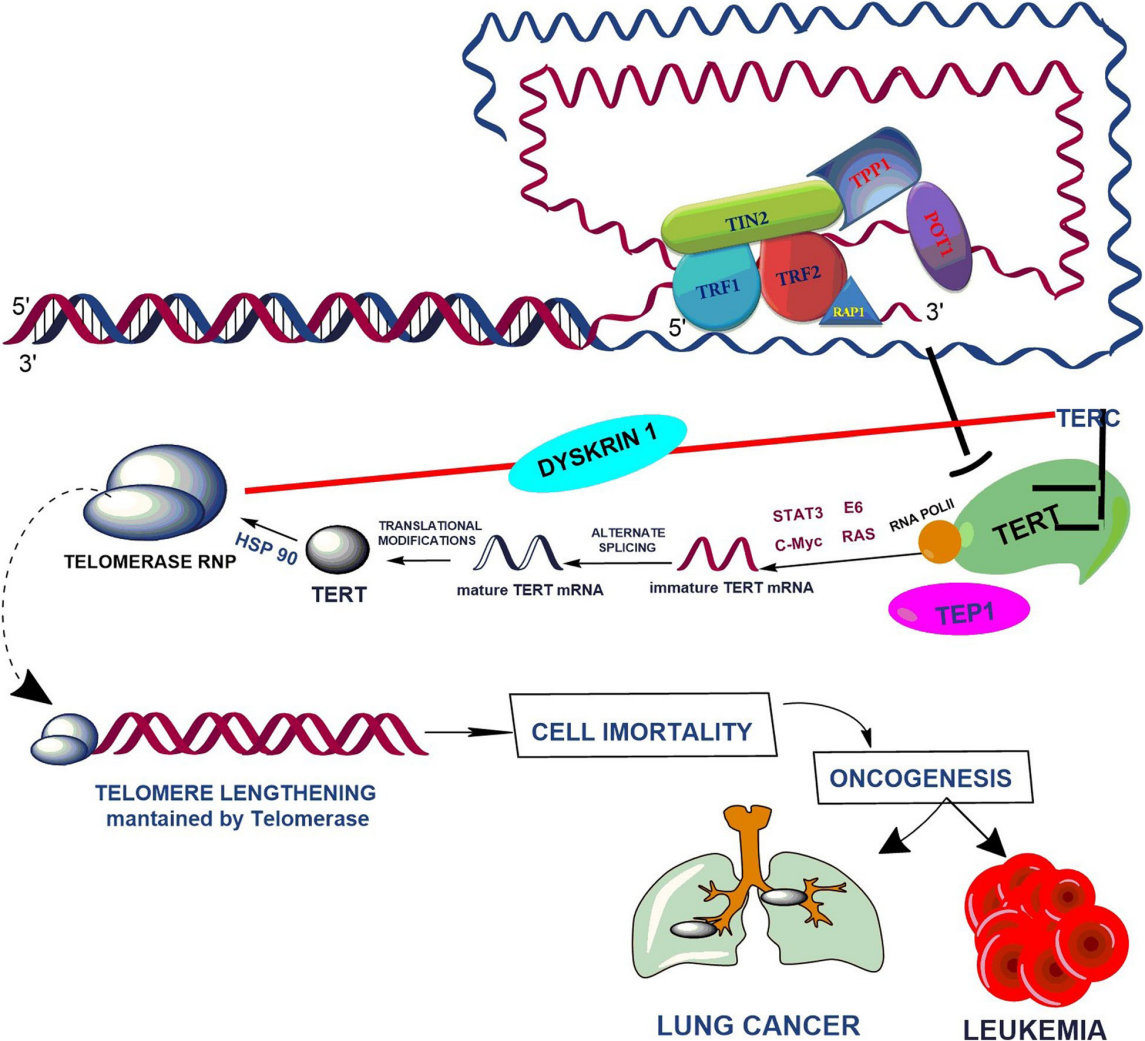
Schematic representation of telomerase regulation and telomere elongation pathway. Molecular alteration leading to overexpression of TERT gene resulting in cell immortality and drive the cell for oncogenesis

Human Pulmonary neoplasms and leukemia’s are heterogeneous in nature, wherein both genetic and non-genetic factors like smoking mainly contribute to its etiology [7]. Jammu and Kashmir is Lung cancer capital of India and Tobacco sales are at highest peak. However, the genetic susceptibility to lung cancer and leukemia is still unclear; the accumulation of low risk alleles may result in inherited risk. Genetic variant rs2853677 of telomere maintenance gene *TERT* is located in intronic region of chromosome 5p15.33. Molecular alteration (polymorphism) in telomere maintenance genes results in destabilization of shelterin complex which could lead to constitutive activation of ribonucleoprotein complex like *TERT* (Fig. 2). The overexpression of *TERT* results in maintenance of telomere length resulting in cell immortality and allows the progression of a preneoplastic lesion towards malignant stages. So these polymorphisms might prove to be a prognostic or predictive biomarker in population group under study.

In order to determine the effect of this SNP rs2385677 on the functioning of *TERT* gene, the bioinformatics in silico analysis of this variant was performed by HSF (v.3.1). It showed that the variant creates broken sites that could influence the physiology of *TERT* gene. Moreover, the splicing effect of the variant needs to be confirmed in-vitro analysis. Although we were not able to fully evaluate the stratified analysis in different subtypes of non-small cell lung cancer and leukemia due to less sample size in each sub type. We also take it as quest to evaluate these subtypes with large sample size in future course. The present study also provides an important information of the genetic etiology of non-small cell lung cancer and leukemia and strengthens clinical findings and highlighting the role of telomere maintenance genes in leukemia and lung cancer, which may have impact to clinical and public health data in population of Jammu and Kashmir. It is also taken into account to screen other telomere maintenance genes in other Indian ethnic groups targeting different cancers [9, 19, 23, 24]. This variant has not been explored with lung cancer or leukemia in any other Indian population groups and this is the first study from the region. These findings could be potential conformations and could be used as potential predictive or prognostic markers in clinical studies of leukemia and lung cancer patients in J&K population groups.

## Conclusion

We observed that the genetic variant rs2853677 in the *TERT* loci is linked with an increased non-small cell lung cancer and leukemia risk in Jammu and Kashmir (North Indian) population. Our result revealed the complex genetic regulation of telomere genetics and highlighted the critical role of telomere maintenance gene (*TERT*) in the pathogenesis of non-small cell lung cancer and leukemia. The limitations of the present study include, the sample size among the subtypes of non-small cell lung cancer and leukemia was relatively small. These findings may also enhance our understanding of inter-population differences in leukemia and lung cancer etiology and strengthens GWAS findings as well, although the physiological functions of these *TERT* SNPs need to be explored in others cancers in future study designs.

## Additional files

Additional file 1: **Table S1.** Showing the genetic association of variant rs2853677 of *TERT* among the various subtypes of non-small cell lung cancer and leukemia. (DOCX 15 kb)

Additional file 2: **Table S2.** Allele frequency distribution and risk associated with smokers and non-small cell lung cancer. (DOCX 16 kb)

## Abbreviations

GWAS: Genome wide association studies
HSF: Human Splicing finder
NSCLC: Non-small cell lung cancer
OR: Odds Ratio
SNP: Single nucleotide polymorphism
TERT: Telomerase reverse transcriptase
