# Supplementary material for: Association of newly identified genetic variant rs2853677 of TERT with non-small cell lung cancer and leukemia in population of Jammu and Kashmir, India
Source: BMC Cancer. 2019 May 24;19:493. doi: 10.1186/s12885-019-5685-2 (PMC6533689; doi:10.1186/s12885-019-5685-2)
Supplement: Supplementary file 1 — Table S1. Showing the genetic association of variant rs2853677 of TERT among the various subtypes of non-small cell lung cancer and leukemia. (DOCX 15 kb) [file 12885_2019_5685_MOESM1_ESM.docx]

**Additional file 1:**

**Table S1:** showing the genetic association of variant rs2853677 of *TERT* among the various subtypes of non-small cell lung cancer and leukemia

| **Genotype** | **Histological Sub types (Non-Small Cell Lung Cancer** | | | **Histological Subtype**  **(Leukemia)** | | | | | **Controls** |
| --- | --- | --- | --- | --- | --- | --- | --- | --- | --- |
|  | **AC**  **(n=112)** | **SCC**  **(n=59)** | **UDC**  **(n=07)** | **ALL**  **(n=35)** | **AML**  **(n=39)** | **CML**  **(n=99)** | **CLL**  **(n=28)** | **MDS**  **(n=02)** | **CONTROLS**  **(n=400)** |
| **AA** | 14 | 7 | 00 | 03 | 04 | 03 | 02 | 00 | 103 |
| **AG** | 58 | 28 | 03 | 17 | 18 | 69 | 14 | 02 | 163 |
| **GG** | 40 | 24 | 04 | 15 | 17 | 27 | 12 | 00 | 134 |
| **TOTAL** | **112** | **59** | **07** | **35** | **39** | **99** | **28** | **02** | **400** |
| **P value** | **0.03** | **0.03** | 0.06 | **0.03** | **0.03** | **.03** | **0.04** | NA | - |
| **Allelic OR** | **1.37**  [1.01-1.86] | **1.55**  [1.04-2.32] | **3.14**  [0.87-11.3] | **1.75**  [1.05-2.80] | **1.71**  [1.04-2.73] | **1.40**  [1.40-1.93] | **1.81**  [1.01-3.22] | NA | - |

Adenocarcinoma (AC), Squamous cell carcinoma (SCC), Large cell undifferentiated Carcinoma (UDC), Acute Lymphoblastic Leukemia (ALL), Acute Myeloid Leukemia (AML), Chronic Myeloid Leukemia (CML), Chronic Lymphoid Leukemia (CLL), Myelodysplastic Syndrome (MDS).
