# Supplementary material for: Association of newly identified genetic variant rs2853677 of TERT with non-small cell lung cancer and leukemia in population of Jammu and Kashmir, India
Source: BMC Cancer. 2019 May 24;19:493. doi: 10.1186/s12885-019-5685-2 (PMC6533689; doi:10.1186/s12885-019-5685-2)
Supplement: Supplementary file 2 — Table S2. Allele frequency distribution and risk associated with smokers and non-small cell lung cancer. (DOCX 16 kb) [file 12885_2019_5685_MOESM2_ESM.docx]

**Additional file 2:**

**Table S2:** Allele frequency distribution and risk associated with smokers and non-small cell lung cancer.

| **GENE/SNP** | **Risk**  **Allele** | **Cases**  **(n=147)** | **Controls**  **(n=70)** | **HWE** | **Allelic OR** | **p Value** | **Dominant OR*** | **p value*** |
| --- | --- | --- | --- | --- | --- | --- | --- | --- |
| *TERT*/rs2853677 | G | G=0.60 | G=0.50 | 0.055 | **1.6**  [1.04-2.3] | 0.03 | **2.4**  [1.1-5.2] | 0.02 |
